# Ethical Issues in the Design and Implementation of Population Health Programs

**DOI:** 10.1007/s11606-017-4234-4

**Published:** 2017-12-18

**Authors:** Matthew DeCamp, Daniel Pomerantz, Kamala Cotts, Elizabeth Dzeng, Neil Farber, Lisa Lehmann, P. Preston Reynolds, Lois Snyder Sulmasy, Jon Tilburt

**Affiliations:** 1 Berman Institute of Bioethics and Division of General Internal Medicine, Johns Hopkins University, Deering Hall 1809 Ashland Avenue, Baltimore, MD 21205 USA; 2 Albert Einstein College of Medicine and Montefiore New Rochelle Hospital, New Rochelle, NY USA; 3 Section of General Internal Medicine, University of Chicago, Chicago, IL USA; 4 Division of Hospital Medicine and Social and Behavioral Sciences, University of California – San Franciso, San Francisco, CA USA; 5 Division of General Internal Medicine, University of California - San Diego, San Diego, CA USA; 6 National Center for Ethics in Health Care, U.S. Department of Veterans Affairs, Washington, DC USA; 7 Harvard Medical School and T.H. Chan School of Public Health, Harvard University, Boston, MA USA; 8 Division of General, Geriatric, Palliative and Hospital Medicine, University of Virginia, Charlottesville, VA USA; 9 Center for Ethics and Professionalism, American College of Physicians, Philadephia, PA USA; 10 Divisions of General Internal Medicine, Health Care Policy & Research, and the Biomedical Ethics Research Program, Mayo Clinic, Rochester, MN USA

## Abstract

Spurred on by recent health care reforms and the Triple Aim’s goals of improving population health outcomes, reducing health care costs, and improving the patient experience of care, emphasis on population health is increasing throughout medicine. Population health has the potential to improve patient care and health outcomes for individual patients. However, specific population health activities may not be in every patient’s best interest in every circumstance, which can create ethical tensions for individual physicians and other health care professionals. Because individual medical professionals remain committed primarily to the best interests of individual patients, physicians have a unique role to play in ensuring population health supports this ethical obligation. Using widely recognized principles of medical ethics—nonmaleficence/beneficence, respect for persons, and justice—this article describes the ethical issues that may arise in contemporary population health programs and how to manage them. Attending to these principles will improve the design and implementation of population health programs and help maintain trust in the medical profession.

Population health, and academic medicine’s role in it, is not new.^1^^,^ 2 However, it is receiving increased attention. The Triple Aim’s goals of improved population health, reduced health care costs, and an improved patient experience of care motivate health system reform.^3^ Other examples include emphasis on population health from national bodies,^4^ efforts to improve quality measures,^5^^–^ 7 and changes to health care financing (e.g., value-based payments).^8^^–^ 10 The Triple Aim may have originated in the United States out of concern for rising health care costs, poorer than expected health outcomes, and a historical absence of focus on population health. However, many of its associated concepts, including the need to reduce health care costs and achieve higher value care, apply to medical practice globally. Many physicians now practice in settings where population health programs affect how care is delivered.

The Institute of Medicine has defined population health as “the health outcomes of a group of individuals, including the distribution of such outcomes within the group.”^11^^,^ 12 Including health equity (i.e., the distribution of health outcomes within the group) in the definition of population health signifies that justice and health disparities are essential considerations, not afterthoughts. In practice, population health is best understood by analyzing the design and implementation of the discrete population health programs (PHPs) undertaken by health care organizations and others for its sake.

PHPs have the potential to improve the health of individuals. Efforts to improve hypertension screening within a population, for example, could result in better treatment of individuals’ hypertension and reduce cardiovascular morbidity and mortality for individuals and the group. Similarly, when PHPs focus on achieving important group outcomes (e.g., hemoglobin A1C targets), many individualsmay benefit.

These potential benefits do not eliminate the need to consider ethical concerns. Proponents of the Triple Aim recognized that achieving the utilitarian (or “greatest good for the greatest number”) goals of the Triple Aim could be constrained by non-utilitarian principles.^3^ Advocates of population health acknowledge that, while good for most patients, most of the time, population health programs may not be in the best interest of every individual in every circumstance.^13^^,^ 14

Physicians practicing within PHPs may face ethical tensions regarding their commitment to individual patients and the broader population.^15^ Population health can push physicians toward traditional public health ethics (where decisions are made with primary concern for large groups of people) and away from clinical ethics (where the decision-making locus is the patient-physician relationship, concerned primarily with individual patient welfare; Table 1).

**Table 1:** How Population Health May Place Physicians in a Zone of Ambiguity Regarding Their Roles

	Public health ethics	Population health ethics	Clinical medical ethics
Unit of concern	Large groups, frequently defined in part by formal city/state/regional/national boundaries	Groups of people, usually defined by where care is received, local geography, and/or payer arrangements	An individual person, defined by the patient-physician relationship
Decision-making locus	Public health agency	Health care organization, system, or payer	Patient-physician relationship
Primary animating ethical principle(s)	Group welfare, safety, or protection from harm		Protection of individual patient welfare (i.e., non-maleficence and beneficence)
Secondary constraining ethical principles(s)	Liberty rights Equity		Respect for individual autonomy/choice Justice
Paradigmatic ethical issues	• Tension between mandatory vaccination or quarantine measures and individual autonomy/choice • How to determine fair resource allocation during an epidemic • Use of behavioral economics to “nudge” food choices and the impact on individual choice	• Tension between improving individual or group performance measures and patient choice regarding their health priorities • Impact of population health activities (e.g., automated reminders, contact from non-care team members) on patient trust in physician • How to appropriately define and respond to “at risk” patients via predictive analytics (including their effect on disparities)	• Respecting patients’ autonomous choices, even when not in their “medical” best interests (informed consent) • Physicians’ obligation to recognize individual- and system-level biases the lead to disparities • Physician engagement in shared-decision making and encouragement of healthy behaviors

Because physicians’ primary ethical commitment remains to individual patients,^16^^–^ 18 physicians have unique roles and obligations regarding population health program implementation. This responsibility is two-fold: first and primarily to fulfill their ethical commitments to the individual patient when practicing under PHPs and secondarily to advocate that health care systems and organizations design and implement PHPs that support these commitments. A prior Society of General Internal Medicine Ethics Committee analysis examined ethical tensions in performance measurement in the context of pay-for-performance programs.^19^ Incentives, financial or non-financial,^20^ add an important ethical dimension to performance measurement. Whether or not costs or incentives are directly involved, however, ethical issues may arise in PHPs that deserve examination. Here we describe these issues and suggest how they might be managed.

## ETHICAL ISSUES IN PHPs

Widely recognized ethical principles—non-maleficence (do no harm) and beneficence (acting in patients’ best interests), respect for persons, and justice—can help identify the ethical concerns that could arise in the design and implementation of PHPs (Table 2).

**Table 2:** Examples of Ethical Issues in Population Health Programs (PHPs) with Example Management Strategies

Ethical questions	Ethical values			
	Non-maleficence and beneficence	Respect for persons	Justice	
			Distributive	Procedural
**Patient level**	“Is this PHP in my individual best interest?”	“Does this PHP protect and enable my choice?”	“Are the benefits and burdens of the PHP shared equally across all patients?”	“Were patients like me involved in the PHP development?”
*Example:*				
Improving the rate of colorectal cancer screening in those age 50–75 by colonoscopy, sigmoidoscopy, or FOBT	Efforts to meet this metric result in unnecessary screening of patients with limited life expectancy as an unitended consequence (e.g., a 74 year old with a terminal illness)	Rather than allowing for discussion of the risks and benefits of colonoscopy, sigmoidoscopy, or FOBT, FOBT is advocated by the PHP as a way to achieve performance quickly	Electronic outreach messages via the EMR preferentially reach certain groups, reducing the potential beneft for others (those with limited English proficiency or without electronic access)	Patients were not involved in the review of the electronic message or its content
Example management strategy	Actively monitor, using EMR data, for evidence of over- and under-screening and take action to prevent it	Present the risks and benefits of possible recommended actions in a balanced way to enable informed choice (not, e.g., encouraging FOBT just to meet the metric quickly)	Ensure messaging is equitably accessible for all groups at the time of initial design (lest these groups be left out, once a goal is met)	Design PHPs with input from patient and family advisory councils (PFACs)
**Physician-level**	“Will this PHP enable me to fulfill my obligations of beneficience to my patients?”	“Do I still feel free to choose and recommend what I believe best for patients?”	“Are the benefits and burdens of PHPs equitably distributed among all clinicians?”	“Were front-line clinicians involved in PHP development?”
*Example:*				
Improving A1C control in patients with diabetes using a new default Diabetes Care Order Set	The focus on diabetes in EMR clinical decision support distracts a physician from other medical or social issues important to individual patients	A pre-filled referral order routes patients to a list of preferred endocrinologists, when the physician and patient believe a different endocrinologist would be best	Primary care phyicians, compared to specialists, bear more of the burden for meeting the metric (e.g., time in EMR documentation)	A diabetes order set is designed without input from front-line physicians, reducing buy-in (and adherence) to it
Example management strategy	Evalute for unintended consequences of PHPs, e.g., whether improvement in one area results in lagging performance in others or reduced patient satisfaction in the encounter	Design referral processes to preserve patient-physician shared decision-making (e.g., informing both about the rationale behind the preferred list and allow exceptions)	Recognize this extra burden by providing adequate time or personnel resources (or, if applicable, shared savings) that result from these efforts	Design the PHP with input from the physicians who will use it to improve buy-in, trust, and PHP success
**Organization-level**	“Will this PHP actually improve the care we deliver to patients, not just improve measurement or documenation?”	“Is the PHP implemented in a way that is respectful? For example is is culturally sensitive and respectful of persons?”	“Are we giving special concern to our most vulnerable patients?”	“What structures ensure ongoing engagement of physicians and patients in the design and implementation of PHPs?”
*Example:*				
Reducing hospital readmissions	A health care organization reduces readmissions to its hospital by shifting care to the emergency room or observation status	A post-discharge home care program is standardized but fails to accommodate patients’ social and cultural differences (e.g., related to family involvement in home care) A medical chart flag for patients at high risk of readmission using predictive analytics is inadvertently stigmatizing	In a setting where certain patients most in need decline post-discharge home care, an organization excludes these patients from its denominator when it calculates its success rate	A post-discharge care management program is designed with patient input, but not from patients who will use the program
Example management strategy	A readmission reduction program pays careful attention to whether it unintentionally shifted costs when it tracks program outcomes	Post-discharge home care programs should be explicitly designed to be respectful of social and cultural differences Careful language should be used to describe “high risk,” preferably crafted with patient input and with training of health care professionals regarding its meaning	Special attention is given to better understanding why patients decline and developing appropriate ways to reach them	Ensure that the patient engagement program that informs program design includes patients representative of the end-user group

### Non-maleficence and Beneficence

Physicians play important public health roles, such as mandatory reporting of certain infectious diseases, and have societal obligations, such as antibiotic stewardship and advocating for health care reform. However, the physician’s primary ethical obligations are to focus on promoting the best interests of individual patients. Non-maleficence requires that physicians ensure PHPs cause no harm to patients. Beneficence requires physicians to support PHPs that actually benefit patients.

In practice, the lines between harmful, non-beneficial, and beneficial effects of PHPs for individual patients may blur. PHPs could improve a population’s overall health while resulting in unintended non-beneficial or even harmful individual consequences. For instance, implementing a standard colorectal cancer screening metric with an age cutoff of 75 years—as might be done with an electronic health record pop-up reminder—appears to have been associated with *underscreening* of healthy individuals over age 75 and *overscreening* of unhealthy individuals under age 75.^21^ Unintentionally, a metric appeared to discourage appropriately individualized clinical decision-making.

Thus, standard recommendations applied rigidly to groups for average benefit may introduce ethical tensions for physicians caring for unique patients. Physicians should support system defaults that apply standard recommendations, but that application must leave space for individualized risk-benefit determination. It should not result in ordering a test to merely “meet the metric” or not ordering a test merely because the metric does not include it.^22^ It might also allow individual patients to choose to participate in a PHP even if they might not individually benefit, assuming that choice is free and informed (e.g., a patient might choose to participate in a population-wide screening program that contributes to organizational population health efforts, even knowing he/she is very low risk and might assume out-of-pocket costs related to screening). Moreover, organizations should develop the capability to monitor for unintended consequences of overuse and underuse.^23^

Beneficence also requires that PHPs support patient-physician relationships, trust, and continuity. PHPs may disrupt trust if patients perceive an overemphasis on certain health conditions (e.g., a focus on diabetes care when the patient is most concerned about other physical or mental health issues) or overly influenced by incentives (e.g., in pay-for-performance^19^^,^ 20 or value-based payment contracts). Trust could also be disrupted if, for example, a patient allergic to the influenza vaccine receives an influenza vaccine reminder and questions why the “doctor forgot me.”

Alleviating tensions between physicians’ obligations to individual patients and population health requires system-level support.^24^ The patient-physician relationship can assist appropriate population health goals,^25^^,^ 26 but this must be done with great care. For instance, personalized outreach letters to patients from their personal physicians may be more effective than one from an unknown clinical director.^27^ Because of the power of this relationship and the potential for its disruption, health systems must ensure that physicians are aware of PHPs, involved in their design, and consulted on the content and timing of patient outreach messaging (particularly when messaging includes individual physicians’ names).

Patient-centered care requires that organizations choose PHPs that truly meet their patients’ needs (whether or not these needs are captured by population health metrics) and build systems that do not erode professionals’ capacity to care for patients.^28^^,^ 29 Organizations must commit to the full spirit, and not just the letter, of population health goals. For instance, when an organization dedicates a PHP to a screening measure (e.g., screening for fall risk), it should simultaneously commit to developing care processes to prevent falls. Similarly, when an organization seeks to reduce readmissions, it should do so meaningfully—not merely by developing workarounds to avoid readmission penalties.^30^^,^ 31

### Respect for Persons and Patient Autonomy

Respect for persons requires protecting a patient’s humanity, including the ability to make free and informed choices about care consistent with the patient’s personal values. This requires recognizing the physician’s clinical judgment in helping patients make these decisions. Informed or shared decision-making—a collaborative, dynamic, and shared process regarding care choices-operationalizes respect for persons.^32^^–^ 34 However, individual patients’ preferences may not align with the preferred option of a given PHP. After all, sometimes the express purpose of a PHP is to influence choice in favor of a particular outcome^35^ and influence physician behavior.^36^

Colorectal cancer screening is again a useful example. Guidelines acknowledge different risks and benefits of screening modalities,^37^ and population health measures are flexible regarding measure requirements.^38^ In fiscal expedience, PHPs could not only encourage fecal occult blood testing (e.g., by making this the default option in an order set or by encouraging it in an outreach letter) but also actively discourage colonoscopy (e.g., by highlighting its inconvenience or cost). If the choice of screening modality is truly “preference sensitive,” doing so may ethically jeopardize patient preferences and individualized clinical judgment. When evidence does not suggest a clearly superior option, it would be more respectful to encourage well-supported and unhurried conversations between physicians and patients (including the use of decision aids, if appropriate)—not stack the deck to favor a particular outcome.^39^

Organizations must design and use PHPs in ways that are evidence-based, align with physician ethical responsibilities, and respect patient preferences and cultural differences. This includes choices related to language, educational materials, and community intervention programs.^40^ For example, post-discharge care programs that accommodate cultural differences are more respectful (and probably more effective at preventing readmissions).^41^^–^ 43 In addition, as organizations increasingly use predictive analytics to identify patients at high risk of admission, readmission, or high utilization of health care, respect requires that these analytics and their display are not stigmatizing (e.g., the use of an airplane EMR icon to flag “frequent flyers,” a pejorative term for “high cost patients”)^44^ and that affected patient populations are involved in analytic design.^45^

### Justice

Both distributive justice (i.e., questions of resource distribution, such as fairly allocating organs for transplantation) and procedural justice (i.e., fair decision processes) must be considered in an ethical analysis of PHPs.

#### Distributive Justice

Emphasis on a fair distribution of resources and explicit protection for vulnerable individuals is found in principles of medical professionalism,^16^ organizational ethics,^46^ and the definition of population health itself.^12^ This consensus requires PHPs to proactively address distributional inequities in the burdens and benefits programs entail. An intervention could conceivably reach an overall measure target (e.g., a 90% influenza vaccination rate) without reaching vulnerable, marginalized, or minority groups (e.g., if the 10% of patients who are unvaccinated includes an overrepresentation of patients in these groups who are especially difficult to reach). Were a PHP to send outreach reminders to patients via an online portal or only in English, it might be an efficient means of outreach but could inadvertently exclude certain patient groups (i.e., those without access to the Internet portal^47^^,^ 48 or those with limited English proficiency). Although individual physicians cannot be held responsible for this systemic shortcoming, physicians’ commitment to justice includes a responsibility to be aware of these potential consequences and advocate for PHPs to proactively tailor interventions and ensure vulnerable, marginalized, or minority groups can share in population health benefits while respecting their right of informed refusal.

Issues of distributive justice are not limited to patients. Because many PHPs focus on primary care, the potential burdens of PHPs in responding to outreach messages, documenting screening, etc., may fall disproportionately on primary care clinicians.^49^ Distributive justice requires health care organizations ensure primary care clinicians have the time, administrative support, and resources to enable them to deliver care to all those in need, including healthy, chronically ill, and disadvantaged populations.

#### Procedural Justice

Procedural justice means that fair decision processes should govern individual and corporate deliberations. Ethically, it requires creating opportunities to engage patients in their own care and in health care organizational decision-making (e.g., as patient members of organizational quality improvement committees or via patient and family advisory councils, PFACs).^50^^,^ 51 Engagement, as desired by the patient,^16^ is therefore part of broader justice-based obligations to create fair decision processes^52^^,^ 53 independent of immediate consequences. PHPs should engage patients and clinicians in their design and implementation. This may lead to better-designed programs that may be more effective and promote greater trust in the organization long-term. As hospitals and health systems create PFACs and other means for engagement, they should do so using available resources and recommended practices.^54^^,^ 55

## Conclusion

Ethical issues that arise in population health are not new. But they may not receive appropriate attention in contemporary fast-paced health care environments where committees and others make decisions regarding population health programming. Physicians play a critical role as individuals and members of a profession when they fuflfill their primary ethical commitment to individual patients, including in the context of population health programs, and advocate that population health programs be consistent with those fundamental ethical commitments.
